# A case report: Upper limb recovery from stroke related to SARS-CoV-2 infection during an intervention with a brain-computer interface

**DOI:** 10.3389/fneur.2022.1010328

**Published:** 2022-11-18

**Authors:** Ruben I. Carino-Escobar, Martín E. Rodríguez-García, Ana G. Ramirez-Nava, Jimena Quinzaños-Fresnedo, Emmanuel Ortega-Robles, Oscar Arias-Carrion, Raquel Valdés-Cristerna, Jessica Cantillo-Negrete

**Affiliations:** ^1^Division of Research in Medical Engineering, Instituto Nacional de Rehabilitación Luis Guillermo Ibarra Ibarra, Mexico City, Mexico; ^2^Electrical Engineering Department, Universidad Autónoma Metropolitana Unidad Iztapalapa, Mexico City, Mexico; ^3^Division of Neurological Rehabilitation, Instituto Nacional de Rehabilitación Luis Guillermo Ibarra Ibarra, Mexico City, Mexico; ^4^Unidad de Trastornos de Movimiento y Sueño (TMS), Hospital General Dr. Manuel Gea González, Mexico City, Mexico

**Keywords:** COVID-19, BCI - brain computer interface, stroke, hemiparesis, case report

## Abstract

COVID-19 may increase the risk of acute ischemic stroke that can cause a loss of upper limb function, even in patients with low risk factors. However, only individual cases have been reported assessing different degrees of hospitalization outcomes. Therefore, outpatient recovery profiles during rehabilitation interventions are needed to better understand neuroplasticity mechanisms required for upper limb motor recovery. Here, we report the progression of physiological and clinical outcomes during upper limb rehabilitation of a 41-year-old patient, without any stroke risk factors, which presented a stroke on the same day as being diagnosed with COVID-19. The patient, who presented hemiparesis with incomplete motor recovery after conventional treatment, participated in a clinical trial consisting of an experimental brain-computer interface (BCI) therapy focused on upper limb rehabilitation during the chronic stage of stroke. Clinical and physiological features were measured throughout the intervention, including the Fugl-Meyer Assessment for the Upper Extremity (FMA-UE), Action Research Arm Test (ARAT), the Modified Ashworth Scale (MAS), corticospinal excitability using transcranial magnetic stimulation, cortical activity with electroencephalography, and upper limb strength. After the intervention, the patient gained 8 points and 24 points of FMA-UE and ARAT, respectively, along with a reduction of one point of MAS. In addition, grip and pinch strength doubled. Corticospinal excitability of the affected hemisphere increased while it decreased in the unaffected hemisphere. Moreover, cortical activity became more pronounced in the affected hemisphere during movement intention of the paralyzed hand. Recovery was higher compared to that reported in other BCI interventions in stroke and was due to a reengagement of the primary motor cortex of the affected hemisphere during hand motor control. This suggests that patients with stroke related to COVID-19 may benefit from a BCI intervention and highlights the possibility of a significant recovery in these patients, even in the chronic stage of stroke.

## Introduction

It is estimated that 1 to 6% of hospitalized patients due to COVID-19 will develop a stroke (1, 2). It has been hypothesized that SARS-CoV-2 infection can create a prothrombotic environment due to an inflammatory response, invasion of vascular endothelial cells and imbalance of angiotensin converting enzyme 2 (ACE2) and renin-angiotensin system (RAS) axis interactions, thus, increasing the risk of ischemic stroke (3). Evidence of stroke related to COVID-19 has been presented as cases. For example, Quenzer et al. (4) reported a large cerebellar stroke in a 32-year-old patient that was associated with severe COVID-19 infection with no initial respiratory symptoms. Rajae et al. (5) described an ischemic stroke in frontal, temporal, and parietal regions presenting as the primary manifestation of COVID-19. Prasad et al. (6) reported a patient that developed ischemic stroke in several vascular territories after recovering from hypoxic respiratory failure due to COVID-19. However, to the authors' knowledge, clinical outcomes, and recovery mechanisms during the rehabilitation process of stroke related to COVID-19 have yet to be reported. Specifically, the neural plasticity mechanisms involved during upper limb motor recovery, one of the main rehabilitation challenges in stroke-related hemiparesis (7), could bring valuable insights for developing rehabilitation strategies for these patients. One promising rehabilitation strategy is comprised by brain-computer interface (BCI) interventions since they have shown evidence of efficacy for the upper limb motor recovery of stroke patients (8, 9). A BCI decodes information from the central nervous system and translates this information into commands for external devices, such as rehabilitation robots (10). In this sense, we report a BCI intervention's clinical and physiological effects in a case of COVID-19-related stroke. The rehabilitation was provided as part of the patient's participation in a clinical trial for stroke neurorehabilitation with a BCI. It is presumed that the presented case is, unique to date since neural plasticity mechanisms during the clinical recovery process in stroke related to COVID-19 are described using transcranial magnetic stimulation (TMS), electroencephalography (EEG), dynamometry, and upper limb clinical measurements.

## Materials and methods

### Patient

**Figure 1:**
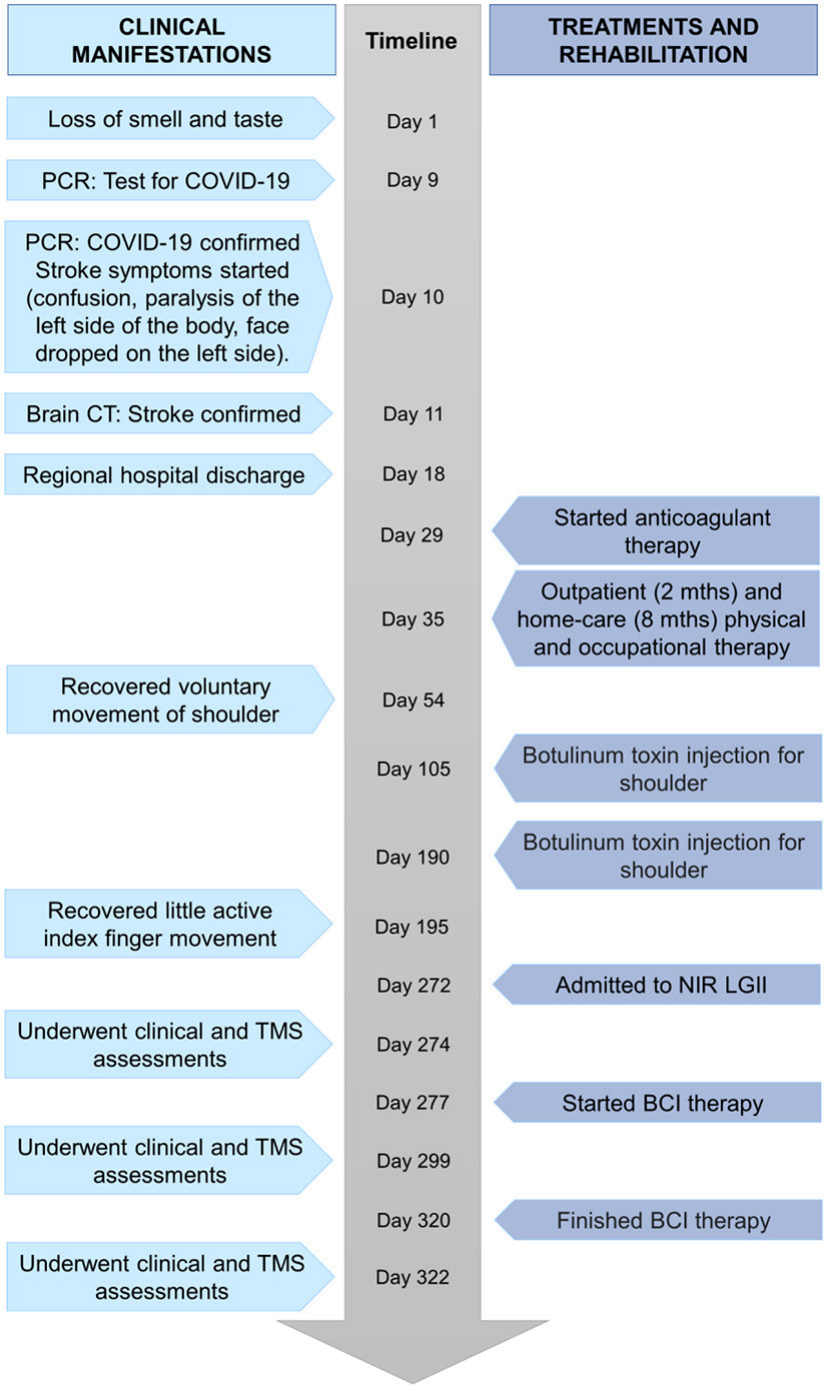
Timeline with relevant clinical information.

**Figure 2:**
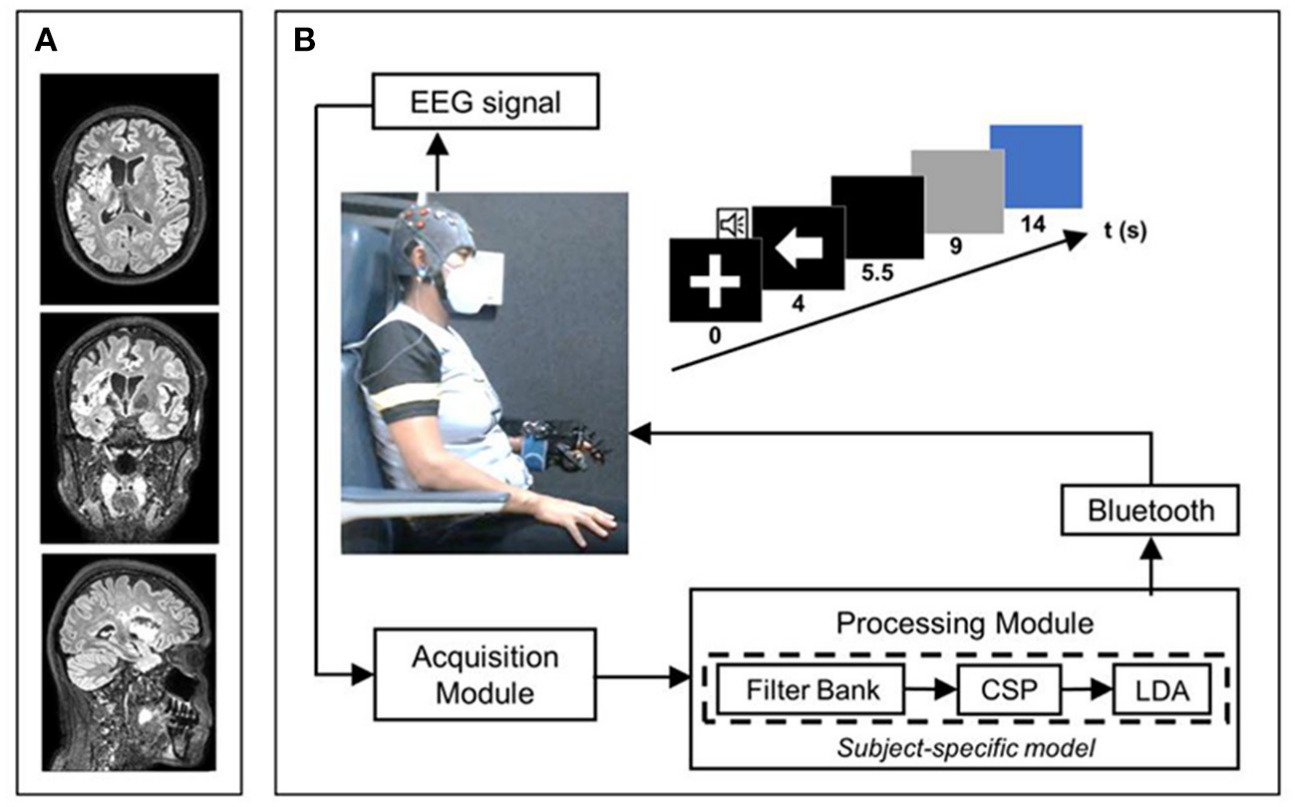
Stroke imaging and depiction of the BCI system. **(A)** Patient's T2 FLAIR MRI sequences were obtained with a 3T Philips Ingenia device at the onset of the BCI intervention. **(B)** BCI system stages and its associated trial timing structure.

A 41-year-old female of Mexican ethnicity, without any previous relevant diseases, presented an ischemic stroke (diagnosed using brain computed tomography) on the same day as being diagnosed with COVID-19 (diagnosed with a PCR test). Her husband, a healthcare worker, had tested positive 7 days earlier for COVID-19. The patient did not have stroke risk factors or previous diagnoses of neurological diseases, was an active athlete at the time of the stroke onset, having completed a dozen marathons, and had not been vaccinated for COVID-19. The only other related COVID-19 symptom before the stroke was the loss of smell and taste. Her blood test results were within normal ranges, including D-dimer. She received acute stroke treatment in the COVID-19 ward of a regional hospital, and after a week was discharged having left hemiparesis. She had outpatient and home-care physical therapy as hemiparesis treatment for 8 months and received two doses of botulinum toxin in her paretic upper extremity as a treatment for spasticity. The main concern of the patient was that she wanted to regain independence lost mainly due to hemiparesis, and that she was not satisfied with her rehabilitation improvement. For these reasons she decided to participate in a clinical trial at the National Institute of Rehabilitation “Luis Guillermo Ibarra Ibarra” (Trial Registry: NCT04724824). The trial aims to evaluate the clinical effects of a BCI therapy for upper limb stroke rehabilitation. Figure 1 shows a timeline with relevant information regarding the episode of care. Figure 2A shows the patient's middle cerebral artery ischemic stroke that comprised the insula, the head of the right caudate nucleus and adjacent white matter.

### BCI intervention

The patient started her participation in the trial after 8 months since stroke onset and was randomly assigned to the experimental group of the clinical trial. Patients in this group underwent a BCI intervention. The Ethical and Research Committees of the National Institute of Rehabilitation “Luis Guillermo Ibarra Ibarra” (Registry Number 25/19AC) approved the research. The patient signed a written informed consent. She received a total of 30 BCI intervention sessions (5 per week, during 6 consecutive weeks). This was the only upper limb therapy administered to the patient during her participation in the clinical trial. The BCI system's acquisition stage was comprised of the recording of 16 channels of electroencephalography (EEG) located in positions F3, FC3, C3, CP3, P3, C5, C1, FCz, Cz, F4, FC4, C4, CP4, P4, C6, and C2, with reference in the right earlobe, and ground in AFz. An amplifier (g.USBAMP, g.tec medical engineering GmbH, Austria) with active electrodes (g.LADYbird, g.tec medical engineering GmbH, Austria) were used for EEG acquisition. The BCI system classified between the motor intention (MI) of the patient's paretic hand (the patient was instructed to attempt to close her fingers to slowly grasp a baseball placed below her hand, without moving her other limbs) and a baseline period in which she was instructed to keep her eyes open while not performing any action. The online processing stage used the filter-bank common spatial pattern algorithm (FBCSP) for feature extraction (11), and linear discriminant analysis (LDA) for classification. A subject-specific model for calibrating the BCI system was computed offline using the data of the previous session. The processing stage is described in the work of Cantillo-Negrete et al. (12). If the system recognized that the patient was performing MI, then it sent wirelessly a command to a robotic hand orthosis that provided passive movement flexion to the paretic hand. In each intervention session, the patient performed 4 runs, each comprised of 20 trials, with every trial containing the temporal sequence described in Figure 2B.

The baseline period comprised the first 4 s of a trial, a white cross was shown on a computer screen during this period and a beeping sound reproduced at the 3^rd^ s indicated to the patient that the MI task was about to begin. After the baseline period, an arrow pointing to the left signaled the patient to initiate the MI of her paretic hand. This arrow was shown for 1.5 s, afterwards, the screen turned black until the 9^th^ s of the trial. The patient was instructed to perform MI during this 5 s period. Windows of 1 s were analyzed by the BCI system processing stage, and if MI was detected during the first 4 s of the MI period, (4^th^ to 8^th^ s of the trial) then for each of these windows detected as MI, the orthosis would perform one-fourth of the maximum flexion displacement of the patient's fingers. Therefore, if MI was detected in all 4 s of the instructed MI period, the patient's fingers were flexed to the maximum displacement capacity of the orthosis. After the 9^th^ s of the trial, the screen turned grey, and the orthosis returned to its original position by performing finger extension. In the 14^th^ s the screen turned blue indicating the patient to relax and move if she needed to, with a random duration of this interval between 4 and 6 s to avoid habituation. BCI success rate in triggering the robotic hand orthosis (i.e., BCI sensitivity) was measured. The system usability scale (SUS) was also measured to assess the user experience with the BCI (13).

### Clinical and physiological outcomes

The Fugl-Meyer Assessment for the Upper Extremity (FMA-UE) and the Action Research Arm Test (ARAT) were measured to record the upper limb motor function (14, 15). The FMA-UE is comprised by 30 items for motor function and 3 items for reflex assessment. Each item must be scored from 0 to 3, with a total score range from 0–66, with a lower score being related with a higher degree of hemiparesis (14). The ARAT is a 19-item scale categorized in the subscales of grasp, grip, pinch, and gross movement, using specially crafted objects for performing each item of the scale. Each item is graded from 0 to 4, and has a total score range from 0–57, with a lower score being associated with the lack of movement of the upper limb (15). The Modified Ashworth Scale (MAS) was used for assessing upper limb spasticity. The MAS is assessed by grading the degree of muscle tone observed while performing flexion and extension movements of the graded limb using a score between 0 to 4 with a higher score related to an increase in muscle tone (16, 17). The Barthel Index (BI) was measured to assess the patient's performance in activities of daily living. It assesses 10 activities of the daily living, including the ability to dress and feed, with a total score ranging from 0 to 100, with a higher score associated with a greater independence (18, 19). The International Classification of Functioning, Disability and Health (ICF) was measured to assess disability of the upper extremity (20), specifically, the b730 item that measures weakness of the hand's muscles was used, graded with an ordinal score from 0 to 4, with the highest score associated with a complete impairment. FMA-UE, ARAT, MAS, BI and ICF were performed by the same rehabilitation physician.

Grip and pinch strength of the paretic limb were separately measured using a Biometrics E-link evaluation system (Hand Grip Dynamometer and Pinchmeter). For each variable, three measurements were acquired, first in the unaffected and then, in the affected hand. Measurements were repeated three times or until the coefficient of variation was below 15% (21).

Corticospinal tract integrity and excitability were evaluated using a transcranial magnetic stimulator (Magstim Rapid^2^, Magstim Co. Ltd., UK) with a figure-of-eight coil. Motor evoked potentials (MEPs) were recorded with electromyography in the first dorsal interosseous muscle of the unaffected and affected hemisphere, in that order, following the procedure recommended for diagnostic transcranial magnetic stimulation (TMS) by the International Federation of Clinical Neurophysiology (22). The resting motor threshold (RMT) was estimated for the patient and afterwards, a cortical excitability curve was calculated from MEPs' amplitudes, at 100%, 120% and 140% of the RMS for both hemispheres (30 MEPs for each intensity), using an automated software (23).

Cortical activity was estimated using event-related desynchronization/synchronization (ERD/ERS). These EEG recordings were planned for evaluating cortical activity during 80 trials per session, in which the patient was instructed to perform hand MI without feedback, using the same cues and trial time structure presented to the patient during BCI intervention sessions. The acquisition was performed using two interconnected g.tec, g.USBAMP devices for recording 32 channels with g.LADYbird active electrodes. In this study, sixteen channels (F3, FC3, C3, CP3, P3, C5, C1, FCz, Cz, F4, FC4, C4, CP4, P4, C6, and C2) were used for the computation of ERD/ERS related to the MI of the patient's paretic hand, to be consistent with the electrodes used for acquiring brain activity with the BCI system during therapies. The preprocessing consisted of 30^th^ order FIR filters, an 8 Hz high-pass filter, a 32 Hz low-pass filter, and a 58 Hz to 62 Hz notch filter, followed by a common average reference spatial filter. Then, a visual inspection was performed to remove trials with excessive artifacts. Afterwards, Complex Morlet wavelets were used to calculate time-varying power in the range of alpha and beta (24) for ERD/ERS computation (25). To assess if there were statistically significant differences (*p* < 0.05) in ERD/ERS across the BCI intervention, a cluster-based permutation test was used. This analysis is based on non-parametric cluster randomization with a multiple comparison procedure (MCP) that has shown higher statistical sensitivity than traditional MCP methods such as the Bonferroni correction (26). All clinical and physiological measurements were acquired at pre-therapy (before BCI interventions), mid-therapy (after 15 intervention sessions) and post-therapy (after the last intervention session).

## Results

Table 1 shows the patient's FMA-UE, ARAT, BI, ICF, and MAS scores throughout the BCI intervention. Upper limb motor function at pre-therapy was limited since FMA-UE scores of 32 to 47 encompass a limited function, as well as scores of 22-42 of ARAT (27). Both FMA-UE and ARAT had gains across the BCI intervention, with an increase of 8 score points for FMA-UE and 24 points for ARAT. The patient had the greatest gain in upper limb motor function in the second half of the intervention. On the other hand, independence for daily living improved from needing help for feeding and dressing/undressing tasks, to being completely independent. The ICF also showed that the patient had a severe upper limb disability that changed to a moderate disability in mid-therapy and further changed to a mild upper limb disability in post-therapy. Moreover, pre-therapy spasticity measured with MAS showed that the patient presented mild spasticity, which changed to an absence of perceived post-therapy spasticity. The affected hand grip strength increased by more than double at post-therapy with the most pronounced increase observed after the first 15 sessions of therapy. Pinch strength also increased during the intervention reaching the twice pre-therapy strength force. At post-therapy, the affected hand had a grip and pinch strength of 25 and 50% of the unaffected hand strength, respectively.

**Table 1:** Upper limb motor function and strength. Higher scores imply less upper limb motor impairment (FMA-UE, ARAT), higher independence for performing activities of daily living (BI), higher upper extremity disability (ICF), or higher spasticity (MAS).

**Clinical score**	**Pre-therapy**	**Mid-therapy**	**Post-therapy**
FMA-UE	45	49	53
ARAT	30	38	54
MAS	1	1	0
BI	90	90	100
ICF	3	2	1
Grip unaffected hand	28.7	30	29.6
Grip affected hand	3.4	8.2	7.8
Pinch unaffected hand	3.5	2.8	3.6
Pinch affected hand	0.9	1.2	1.8

Upper limb strength was measured using dynamometry (kgf) in the affected and unaffected hands.

**Figure 3:**
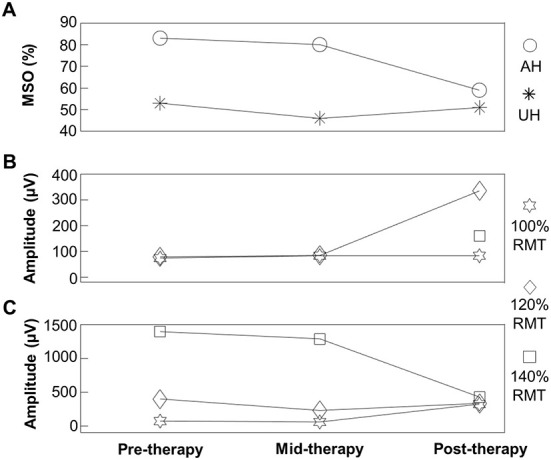
**(A)** Resting-state motor threshold (RMT) for the affected (AH) and unaffected (UH) hemispheres. **(B)** Motor evoked potential amplitude in the affected hemisphere (AH) at 100%, 120%, and 140% of the RMT at pre-therapy, mid-therapy, and post-therapy. **(C)** Motor evoked potential amplitude in the unaffected hemisphere (UH) at 100, 120, and 140% of the RMT at pre-therapy, mid-therapy, and post-therapy.

Figure 3 shows the patient's RMT of each hemisphere, obtained using TMS, as well as MEP amplitudes at 100%, 120%, and 140% of the RMT along the BCI intervention. It can be observed that the RMT of the affected hemisphere lowered slightly from 83 to 80% at mid-therapy. However, at post-intervention, became lower, reaching 59% of the maximum stimulator output. The RMT in the unaffected hemisphere remained stable across the intervention with 53, 46, and 51% of the maximum stimulator output at pre-therapy, mid-therapy, and post-therapy, respectively. MEPs amplitude in the affected hemisphere could only be measured at 140% of the RMT at post-therapy due to 140% of the RMT surpassing the maximum possible stimulator output in the pre-therapy and mid-therapy measurements. In the unaffected hemisphere, MEPs amplitude was lower at post-therapy reaching 427 μV at 140% of the RMT compared to the pre-therapy and mid-therapy measurements that presented higher amplitudes, 1,390 μV and 1,286 μV, respectively, at 140% of the RMT.

**Figure 4:**
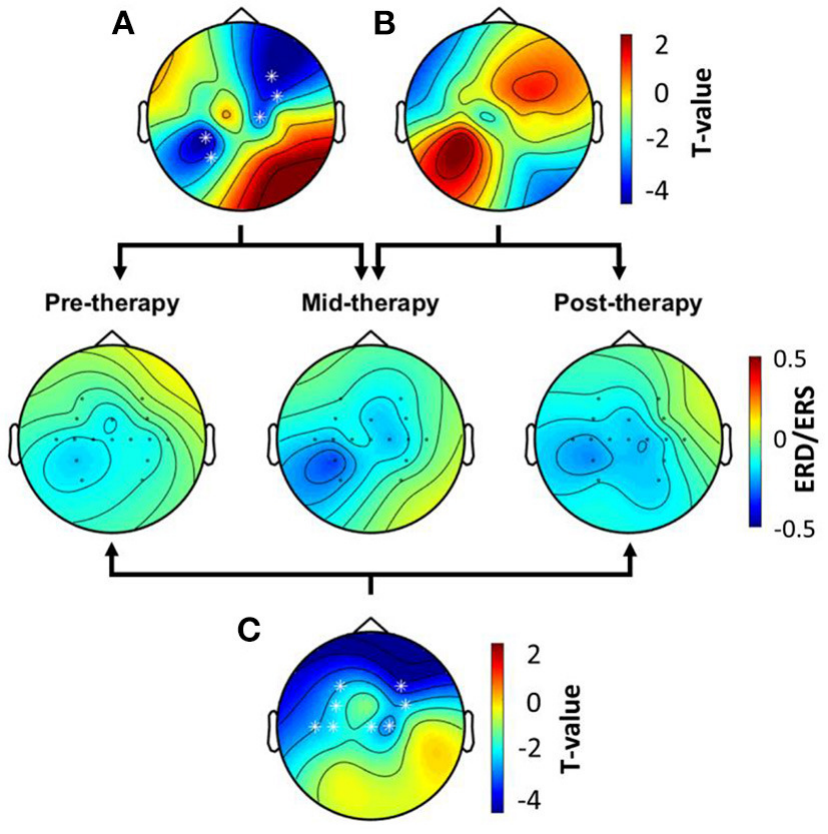
Topographic maps showing ERD/ERS over time. **(A)** Significant ERD/ERS differences between pre-therapy and mid-therapy assessed with the cluster-based permutation test. **(B)** Significant ERD/ERS differences between the mid-therapy and post-therapy. **(C)** Significant ERD/ERS differences between the pre-therapy and the post-therapy. Significant clusters across therapy stages are marked with white asterisks.

Figure 4 shows the topographic ERD/ERS maps across the intervention. It also shows significant differences computed with the cluster-based permutation test between intervention recordings. During pre-therapy, cortical activations shown as ERD were observed in the unaffected hemisphere. However, at mid-therapy and post-therapy, ERD was also elicited in the affected hemisphere in electrode C2 located over the primary motor cortex. Significant clusters implied that differences of more pronounced ERD were observed in both the affected and unaffected hemispheres, which was observed at mid-therapy, and remained post-therapy.

The BCI success rate was 61.3%. This shows that the patient was able to successfully activate the robotic orthosis with the MI of her paralyzed hand in more than half of the time during the performed attempts. The SUS score reported by the patient was graded as 92.5 out of 100 points, with this score being in the range of the “Best Imaginable” user experience (28).

## Discussion

The patient had a good adherence to the intervention, measured by an attendance to all the 30 sessions of therapy, and good tolerability, since she was able to complete 80 trials of the MI task, in every single BCI session. There were no adverse or unanticipated events during the BCI intervention. After the BCI intervention, the patient had an upper limb motor recovery above the minimal clinically significant difference of 5.25 points for FMA-UE (29). In addition, the recovery measured with ARAT was more than 4 times the minimal clinically significant difference of 5.7 points (30), implying that the patient presented an improvement from a limited to a notable upper limb motor function (27). This degree of recovery is not commonly observed in chronic stroke patients. For example, Dromerick et al. reported an improvement of 2.41 ± 2.2 points of ARAT after a task-specific motor intervention (31), and Ackerley et al. reported an improvement of 2 points of ARAT after 1 month of intermittent theta-burst TMS treatment (32). Moreover, the recovery of the patient was higher than the reported in stroke populations that underwent the BCI intervention reported by Ramos-Murguialday et al. (33) with a gain of 3.4 ± 2.2 points of FMA-UE, and by Frolov et al. (34) with an average gain of 5 points of FMA-UE and 2 points of ARAT. Furthermore, according to the BI there was an improvement in the performance of activities of daily living that require the use of the upper extremities, such as feeding and dressing, and a reduction of the disability shown in the upper extremity measured with ICF. Also, spasticity was reduced to the point of being undetectable after the BCI intervention, which is in line with a reported association between a lower degree of spasticity and a higher upper limb motor function (35). Therefore, the patient presented a significant clinically measured recovery that was noticeable after the BCI intervention.

Upper limb strength and corticospinal excitability also showed differences across the intervention. Grip and pinch strength doubled, which was within the range of the recovery observed in moderately impaired stroke patients after a high-intensity upper limb-focused therapy for 6 to 12 weeks (36). On the other hand, corticospinal excitability increased in the affected hemisphere, while it decreased in the unaffected hemisphere after the intervention, which has been described as a mechanism of stroke recovery (37). Furthermore, the high degree of observed recovery, seen in patients that do not need to recruit secondary motor regions such as the dorsolateral premotor cortex or the supplementary motor cortex (38), coupled with the enhanced cortical excitability in the affected hemisphere, allow suggesting that recovery mechanisms involved the primary motor cortex. Cortical activations computed from EEG also support this hypothesis, since electrodes located over the primary motor cortex of the affected hemisphere recorded a significantly enhanced activity after the intervention. This is important since enhanced cortical activity over the affected hemisphere's primary motor cortex has been associated with a significant recovery of upper limb motor function (39). Therefore, the neuroplasticity mechanism that was involved in the recovery of the patient's upper limb motor function, was the reengagement of the primary motor cortex for movement control, which allowed to reestablish the functional integrity of the affected hemisphere's corticospinal tract.

Stroke and COVID-19 have been associated across several studies (1, 2, 4, 5, 40), even in young patients of 33 to 49 years of age with a low prevalence of risk factors, as was the case with the patient in the study (41). Interestingly, the present case shared features with a patient from a case series reported by Diaz-Segarra et al. (42) including being a young patient, having non-severe COVID-19, being diagnosed on the same day of presenting the stroke with COVID-19, having a mid-cerebral artery stroke, being discharged after a week from hospitalization, and presenting hemiparesis. Hence, young patients that present a stroke associated with non-severe COVID-19 could be potentially a new stroke subgroup in which rehabilitation effects have not been previously studied. It is possible that the young age of these patients, combined with the recovery of COVID-19 and the associated decrease of the thrombotic environment observed during the disease, could make possible the promotion of neuroplasticity mechanisms during rehabilitation regimes, even in the chronic stage of stroke. Experimental therapies, such as those based on BCIs could be a potential complementary intervention in COVID-19-related stroke, which is supported by the significant degree of upper limb motor recovery observed in the present case. Furthermore, the patient's high degree of recovery was observed only after the intervention with the BCI system during the chronic stage of stroke. This is remarkable since this amount of recovery could have been more likely in the subacute stage, in which the patient received physical therapy, due to spontaneous recovery mechanisms that are hypothesized to be responsible for most of the motor gain observed in stroke (43, 44). Thus, implying that the BCI intervention was directly associated with the significant observed stroke recovery. A novelty of the present study is that it shows it is possible for a patient with a COVID-19-related stroke to reengage their lesioned hemisphere, shown by an increase in the cortical excitability of this hemisphere, while recovering upper limb motor function. This provides insights into similar recovery mechanisms compared to stroke of other etiologies and highlights the importance of acquiring physiological measurements such as TMS, EEG, and dynamometry for assessing recovery mechanisms in stroke, which are not all reported in BCI stroke interventions (8, 34, 45, 46). Furthermore, a strategy for improving therapies for these patients, and for looking at specific physiological mechanisms can be derived for being used in further interventions regarding stroke related to COVID-19. Also important, is that the patient achieved an acceptable success rate, which is within the range of the performance reported in a BCI intervention for stroke (34). Although BCI performance using MI tasks by stroke patients has been reported, it has not been previously reported in stroke related to COVID-19 implying that a BCI system can be controlled by these patients. In addition, the patient reported to felt comfortable using the BCI, implying that the degree of complexity of the system is adequate for stroke patients.

The present study has limitations that need to be acknowledged. The first one is that one case is presented, not allowing to fully infer the clinical effects of the BCI intervention in stroke related to COVID-19. However, to the authors' knowledge, this is the first report of the upper limb rehabilitation in stroke related to COVID-19 spanning most of the patient's rehabilitation process, and it provides for the first-time evidence that a significant recovery of the upper limb motor function is achievable. This is important since several studies have highlighted the significance of assessing complete rehabilitation scenarios of these patients (40, 42, 47). Another limitation is the assessment of a single possible stroke related to COVID-19 subgroup, comprised of young patients that have at least some degree of preserved corticospinal integrity in their affected hemisphere. Other stroke-related COVID-19 subgroups should also be analyzed in rehabilitation scenarios. Nevertheless, it is likely that the presented case will provide valuable information for the upper limb rehabilitation and neuroplasticity processes in stroke related to COVID-19 and could aid in the development of new complementary rehabilitation strategies using BCI systems.

## Conclusions

A significant upper limb motor recovery was possible in the chronic stage of stroke related to COVID-19 using an experimental BCI intervention. The main neuroplasticity mechanism associated with this recovery was the reengagement of the primary motor cortex for upper limb control. Although a particular case is presented, it provides evidence that young patients with stroke related to COVID-19, with measurable corticospinal excitability in the affected hemisphere, may have a good degree of recovery with BCI-based experimental interventions.

### Patient perspective

“I am very grateful to the team that made this research possible, today I open my hand with greater control, I can give a real hug to each member of my family. I can ride a bicycle, since I am able to break and control the steering wheel better, this has made me very happy. I have recovered part of my life, self-esteem and even faith.”

## Data availability statement

The raw data supporting the conclusions of this article will be made available by the authors, without undue reservation.

## Ethics statement

The studies involving human participants were reviewed and approved by Research and Ethics Committees of the National Institute of Rehabilitation “Luis Guillermo Ibarra Ibarra”. The patients/participants provided their written informed consent to participate in this study. Written informed consent was obtained from the individual (s) for the publication of any potentially identifiable images or data included in this article.

## Author contributions

JC-N, RC-E, AR-N, and OA-C conceived and designed the study. JC-N, RC-E, MR-G, AR-N, JQ-F, and EO-R performed data collection. JC-N, RC-E, MR-G, and RV-C analyzed the data. JC-N, RC-E, and OA-C drafted and edited the manuscript. JQ-F, AR-N, EO-R, and RV-C provided critical revisions. All authors have approved the final version of the manuscript submitted for publication.

## Funding

This work was supported by Consejo Nacional de Ciencia y Tecnología (CONACYT) [SALUD-2018-02-B-S-45803].

## Conflict of interest

The authors declare that the research was conducted in the absence of any commercial or financial relationships that could be construed as a potential conflict of interest.

## Publisher's note

All claims expressed in this article are solely those of the authors and do not necessarily represent those of their affiliated organizations, or those of the publisher, the editors and the reviewers. Any product that may be evaluated in this article, or claim that may be made by its manufacturer, is not guaranteed or endorsed by the publisher.
